# Moving into the mainstream: healthcare professionals’ views of implementing treatment focussed genetic testing in breast cancer care

**DOI:** 10.1007/s10689-019-00122-y

**Published:** 2019-01-28

**Authors:** Nina Hallowell, S. Wright, D. Stirling, C. Gourley, O. Young, M. Porteous

**Affiliations:** 1 Wellcome Centre for Ethics and Humanities and the Ethox Centre, Nuffield Department of Population Health, Big Data Institute Li Ka Shing Centre for Health Information and Discovery, University of Oxford, Old Road Campus, Oxford, OX3 7LF UK; 2 Usher Institute of Population Health Sciences and Informatics, University of Edinburgh, Edinburgh, UK; 3 MRC Institute of Genetics and Molecular Medicine, University of Edinburgh, Edinburgh, UK; 4 Cancer Research UK Edinburgh Centre, MRC Institute of Genetics and Molecular Medicine, University of Edinburgh, Edinburgh, UK; 5 Edinburgh Breast Unit, Western General Hospital, Edinburgh, UK

**Keywords:** *BRCA1* and *BRCA2* treatment focussed testing, Mainstreaming, Clinician perspectives, Qualitative analysis

## Abstract

A proportion of breast cancers are attributable to *BRCA1* or *BRCA2* mutations. Technological advances has meant that mutation testing in newly diagnosed cancer patients can be used to inform treatment plans. Although oncologists increasingly deliver treatment-focused genetic testing (TFGT) as part of mainstream ovarian cancer care, we know little about non-genetics specialists’ views about offering genetic testing to newly diagnosed breast cancer patients. This study sought to determine genetics and non-genetics specialists’ views of a proposal to mainstream *BRCA1* and *2* testing in newly diagnosed breast cancer patients. Qualitative interview study. Nineteen healthcare professionals currently responsible for offering TFGT in a standard (triage + referral) pathway (breast surgeons + clinical genetics team) and oncologists preparing to offer TFGT to breast cancer patients in a mainstreamed pathway participated in in-depth interviews. Genetics and non-genetics professionals’ perceptions of mainstreaming are influenced by their views of: their clinical roles and responsibilities, the impact of TFGT on their workload and the patient pathway and the perceived relevance of genetic testing for patient care in the short-term. Perceived barriers to mainstreaming may be overcome by: more effective communication between specialities, clearer guidelines/patient pathways and the recruitment of mainstreaming champions.

## Introduction

The contribution of *BRCA1* and *BRCA2* mutations to the incidence of breast and ovarian cancer has been acknowledged for a number of years [1, 2]. Cumulative lifetime risks (until age 80 years) of breast cancer associated with *BRCA1*/*BRCA2* mutations are estimated to be as high as 72% (65–79%) and 69% (61–77%), respectively, while ovarian cancer risks are 44% (36–53%) and 17% (11–25%) [3]. Patients with breast cancer who have a germline *BRCA1*/*BRCA2* mutation are at increased risk of ipsilateral [4] and contralateral tumours compared with those presenting with sporadic disease [3]. Genetic testing of cancer patients and their unaffected relatives facilitates the implementation of risk-reducing strategies including: enhanced surveillance, chemoprevention and risk-reducing surgery (bilateral mastectomy and/or bilateral salphingo-oophorectomy) [5].

Recent technological advances in sequencing, decreasing costs and the development of new treatments, for example, poly(ADP-ribose) polymerase inhibitors (PARPi), mean that now *BRCA* testing can be used to inform cancer treatment plans [6]. Knowledge of *BRCA* mutation status of breast cancer patients can inform the extent of breast surgery and the appropriateness of adjuvant radiotherapy for those considering risk-reducing mastectomy and (neo-)adjuvant chemotherapy regimen [7–9]. Ovarian cancer patients are now selected for treatment with the PARPi olaparib based on their *BRCA* mutation status and their response to first line therapies [6, 10].

Despite the fact that *BRCA* testing has been available for over two decades, diagnostic testing has been limited to women with a strong family history, plus specific tumour characteristics, and it is only more recently that treatment focussed *BRCA* genetic testing (TFGT) has become more widely available for newly diagnosed cancer patients, leading to the possibility of mainstreaming this service in oncology.

### Mainstreaming genetics/genomics

Mainstreaming, namely, the implementation of genetic/genomic testing in other specialities, for example, oncology, to aid diagnosis and/or treatment, offers the promise of streamlined pathways and tailored treatment for individual patients [11, 12]. A number of challenges to the implementation of mainstreamed genetic services in the UK have been identified, including: a lack of consistency in services and patient management including the interpretation of genetic variants, the educational requirements of non-genetics specialists who may be required to offer testing, a lack of funding and human resources within clinical genetics to support mainstream services plus a lack of pre-existing information, guidelines or protocols [13, 14]. Despite these challenges, there is evidence that mainstreaming of *BRCA1* and *2* testing in gynaecological-oncology clinics in both the UK and Australia has been successfully implemented [15–17]. However, there is a lack of data on the impact of mainstreaming of *BRCA* testing in breast clinics.

A recent study suggests approximately a third of newly diagnosed breast cancer patients in the US are not offered *BRCA1*/*BRCA2* genetic tests, despite the fact that the result may inform their treatment [18]. This may be due to the fact that testing for *BRCA2*/*BRCA1* mutations is perceived as more informative for prevention than determining treatment options in breast cancer [19], although recent research on the *BRCA1*/*BRCA2* mutation carriers’ response to carboplatin therapy [9] suggest this perception may change. Indeed, earlier Australian studies have suggested that healthcare professionals (oncologists, breast surgeons and breast care nurses) do regard *BRCA* testing as potentially valuable in the management of breast cancer as well as having a positive impact on risk management decisions, with the majority of respondents suggesting this service should be mainstreamed [20, 21].

It is therefore, more likely that the failure to implement TFGT in mainstream breast cancer care results from the existence of a knowledge or skills gap. A recent US study found that breast surgeons, particularly those who see fewer patients, report they lack confidence to discuss *BRCA1* and *BRCA2* testing with patients [22]. A UK based study similarly suggests that non-genetics specialists (breast surgeons, medical and clinical oncologists) question their ability to correctly interpret genetics reports, although the breast surgeons in this study rated themselves as more confident about interpreting reported genetic variants than the medical oncologists [23]. One of the problems of many of these studies is that the health professionals involved had little, if no, experience of offering TFGT. To address this issue we undertook a study of UK genetics and non-genetics healthcare professionals’ perceptions of the delivery of TFGT. This paper describes their views of a proposal to mainstream this service in either the breast or oncology clinic at the study site.

## Methods

### TFGT at the study site

**Fig. 1:**
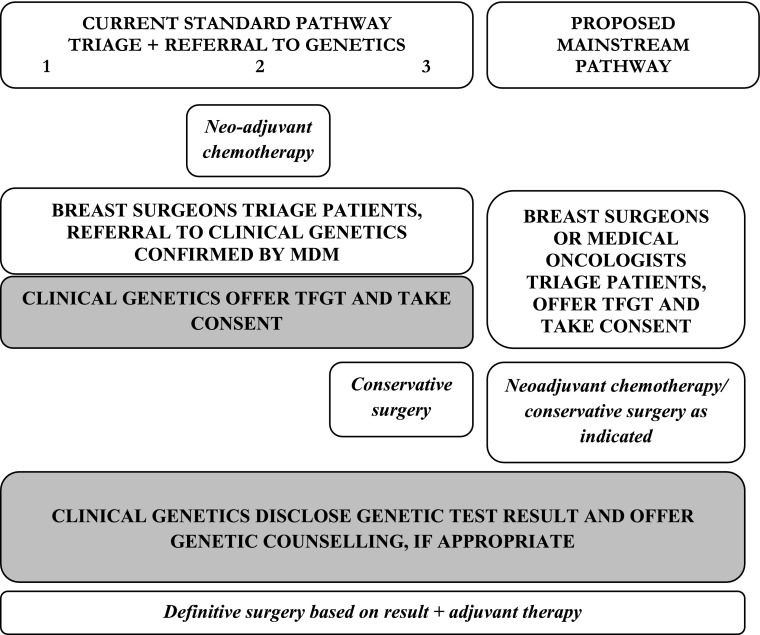
Current and proposed breast cancer care pathways at study site. Shaded area indicates tasks undertaken by clinical genetics team

This study was based in a tertiary referral centre that offers TFGT to newly diagnosed patients with ovarian or breast cancer. When the study began patients with ovarian cancer were offered, and consented for TFGT by their gynaecological-oncologists in a mainstreamed pathway, while newly diagnosed breast cancer patients were triaged by breast surgeons and referred to clinical genetics for TFGT (see Fig. 1). Triage of breast cancer patients considered a combination of factors: age at diagnosis (< 40 years), tumour type (triple negative) plus a family history of disease. The multidisciplinary team meeting (MDM) confirmed onward referral following discussion of individual cases. TFGT was implemented at three different points in the breast care pathway depending on disease presentation, either: prior to any treatment (Pathway 1), following neoadjuvant chemotherapy (Pathway 2) or following conservative breast surgery (Pathway 3). In all cases, decision-making about risk-reducing mastectomy took into account *BRCA* mutation results. In addition to taking consent for TFGT, the clinical genetics team disclosed results and discussed the personal and familial risk implications with those identified as carrying a pathogenic mutation or Variant of Uncertain Significance, and initiated a familial cascade, if appropriate.

During this study it was decided that TFGT should be mainstreamed in the breast cancer pathway and the clinical genetics team began discussions with the surgical and medical oncology teams to decide who would assume responsibility for offering and consenting breast cancer patients for genetic testing, with a view to piloting a mainstreamed service during the summer of 2018 (see “Results” section). As indicated in Fig. 1, while the clinical genetics team were prepared to outsource offering and consenting patients for genetic testing to streamline patient care, they proposed to retain responsibility for results disclosure (to patients and breast cancer specialists), genetic counselling and familial cascading.

### Recruitment, data collection and analysis

All staff responsible for discussing and/or (potentially) offering TFGT in the breast, oncology and genetics clinics at the study site were emailed a recruitment pack (invitation letter, study information sheet and expression of interest form). In addition, SW described the study to clinicians attending the breast cancer MDMs.

Qualitative data were collected during in-depth face–face interviews; these were informed by a topic guide and digitally recorded. Interviews focused on: staff experiences and views of TFGT and its role in clinical practice, perceptions of mainstreaming of genetic testing and ethical implications of TFGT. Interviews took place in the hospital and lasted 19–77 min. Digital audio files were transcribed verbatim. SW and NH independently reviewed and coded interview transcripts using NVivo11 software (QSR International Pty Ltd., 2015). Codes and larger themes were inductively and deductively determined from the interviews and literature, respectively [24]. Below we report on two themes in the analysis ***Staff perceptions of mainstreaming*** and ***Moving genetic testing into the mainstream***.

## Results

Twenty-two of the 31 eligible staff members involved in (potentially) offering TFGT at the study site accepted the invitation, including: 7/12 Breast surgeons (58%), 6/10 medical oncologists (60%) and 6/7 (86%) members of the clinical genetics team. Twenty-one interviews were undertaken between February 2017 and January 2018, only data collected in interviews with the 19 staff members (clinical genetics, oncology and surgical teams) involved in the care of breast cancer patients are reported here. The data suggest that staff views of mainstreaming were influenced by perceptions of: their *role responsibilities*, its *workload* implications and the perceived *relevance* of TFGT for their practice.

### Genetics team’s perceptions of mainstreaming

The clinical genetics team (see Table 1) described their primary role as facilitating individuals and families to make decisions about their genetic risks and risk management. Bearing this in mind, the introduction of TFGT in breast cancer care at the study site has had a major impact on the clinical genetics team. Team members said that the introduction of TFGT had increased their workload and that they had put on extra clinics to consent newly diagnosed patients for TFGT. They worried that having a referral to clinical genetics for TFGT at the point of diagnosis could be anxiety-provoking for patients. Consequently, with the aim of improving patient care, the team had agreed to invite the surgical team and the medical oncologists at the study site to consider taking on the task of offering TFGT and consenting their patients. Team members observed that this potential change in service provision would not only be in patients’ interest, but also would allow them to concentrate on offering genetic counselling and familial cascading to the smaller group of patients identified as carrying a pathogenic mutation. Thus, as far as the genetics team were concerned, mainstreaming TFGT would streamline the patient pathway and provide better patient care by ensuring that only those patients who need genetic services would receive them.

**Table 1:** The clinical genetics team’s perceptions of TFGT

**Role responsibility: Facilitating individual and familial decision-making**
*In genetics we’re like acutely aware of families and, other people who are at risk, the implications of your test for your family CG5*
*…if testing is done through, say for the breast patients through the breast unit by the breast clinicians, the genetics services then only have to engage with those with an underlying genetic condition, so perhaps genetics services could be more focused on the patients that need that service. CG3*
**Redesigning the care pathway: Providing the best service**
*As professionals we have to look at the best service for the patients, whether it’s having genetic discussion and testing at the point of diagnosis or whether they are referred on to our service, that’s obviously the most important thing. I think as professionals we have the skills to pick up someone coming along with a positive gene test and take them on their journey rather than starting their journey with them pre-test…I think the things to take into consideration are sort of speed, if speed is what people want, and also the practicalities, take into consideration the practicalities of the lady who has maybe a distance to travel from the hospital she’s been diagnosed at, if she then has to return to that hospital two days later for another consultation you could perhaps take that into consideration, the literal practicalities of the genetic testing…. CG2*
*Patients are getting referred at the point of diagnosis, and it’s meant that we’ve had clinics put aside on a weekly basis that are for patients who’ve just got a diagnosis, …. usually the patient just needs half an hour for that consultation CG1*
**Relevance for practice: Less relevant for clinical genetics practice**
*if it’s being done for treatment implications it’s therefore a diagnostic test. And it perhaps shouldn’t be considered any differently from any other diagnostic test that you would do if there are true treatment implications for that person. GC3*
*I think there’s an assumption that treatment is going to become a bigger and bigger thing, that we’re seeing the start of treatment being influenced by genotype, so in a way it’s partly, it’s a good place to start, isn’t it. I think that women are also, there’s stuff out there sort of suggesting that it might change their treatment, and if you were going through breast cancer you might think, oh actually I need to know that. It’s come out of trying to do genetic counselling in a woman that’s just been diagnosed with breast cancer and how the counselling model doesn’t fit so well with somebody that’s got all that other stuff going on and it actually sits neater in the oncology model. CG4*

### Surgical team’s perceptions of mainstreaming

Most members of the surgical team said they were happy with the role they currently play in TFGT, namely triaging patients for referral to clinical genetics, and were not keen to implement a mainstreamed service in the breast clinic. First, they said they lacked the requisite expertise to counsel patients about genetic testing (see Table 2). Many interviewees reflected that a surgeon’s primary responsibility is to treat patients and “get the cancer out” as quickly as possible, not to talk to women about genetics. Second, surgical team members commented that they had neither the time nor the capacity to take on another task. Many talked about their unsustainable workload and a couple drew explicit comparisons with the genetics clinic, which they suggested had a much smaller patient list than the breast clinic.

**Table 2:** The surgical team’s perceptions of TFGT

**Role responsibility: Treating cancer**
*We’re there to diagnose their lumps and treat them. But in the middle of the thing we’re being asked to do all this stuff TFGT as well. And I think that’s becoming pretty difficult to do. ‘Cause we’re mainly interested if you come with a lump. You want to know what your lump is. We’d want to do all the investigations that day, work out what it is. And that’s what the focus is. S2*
*The genetics team wanted us to do all this this rapid testing. It’s because they’re overwhelmed, they just want to get us to spend the time talking to the patients about it. Yeah of course we want more patients assessed than we used to, for obvious reasons, but we don’t have the time or skills to counsel people about gene testing. We wouldn’t dream of asking a geneticist to counsel people about breast surgery S6*
**Redesigning the care pathway: Mainstreaming TFGT will increase workload beyond capacity**
*When you looked at the numbers it mainstreaming was going to add up to hundreds of hours a year that we just don’t have time or staff to do. Which is exactly why they genetics wanted us to do it, because they don’t have the time or staff to do it either S5*
*We had about 35 patients in our clinic this morning between two people. In my afternoon follow-up clinic I’ve got 14, 16 patients coming between two and half-four or something, so you cannot stop for 40 min to have a chat with somebody about consenting for gene testing on top of everything else you’re doing. S3*
**Relevance for practice: TFGT less relevant for surgical practice**
*Quite often we feel that the priority is treating the cancer. So we could do a smaller surgical procedure to deal with the cancer initially. The patient could then have chemotherapy if appropriate. And then if the genetic testing showed that they carried a mutation, then we could consider more radical surgery later on. S1*
*It’s the cancer, treating the cancer should be the first priority. Because when you know that the patient’s BRCA, surgically what you are doing is prevention, not treatment. So the first thing is to treat the cancer, the prevention of the secondary cancer, prevention of recurrence can be done, and then what you try to achieve is the best cosmetic result. S4*

Finally, from the surgical team’s perspective, TFGT is not regarded as having much relevance for treatment decision-making. While they acknowledged that in some cases a woman’s mutation status will inform the extent of her surgery, or the way it is performed, in most instances TFGT, which many characterised as revealing future risks, is perceived as clashing with surgical priorities (Table 2 e.g. S1). As far as the surgeons where concerned, treating actual disease has priority over risk management or disease prevention. Indeed, some talked about the potential difficulties of fitting TFGT into the timeline dictated by the UK’s NHS treatment targets.

### Oncologists’ perceptions of mainstreaming

In contrast to the surgical team, the medical oncologists responded positively to their proposed involvement in mainstreaming. Offering TFGT and consenting newly diagnosed patients did not concern the oncologists who reported feeling confident that, given their current role and responsibilities, they had more than enough expertise to take on this task. Indeed, many reflected that they currently have to present and explain a range of different types of complex treatment and prognostic information to patients and families and therefore, explaining the risks and uncertainties of genetic testing would not be particularly challenging (Table 3).

**Table 3:** Oncology team’s views of TFGT

**Role responsibility: Diagnosing and treating cancer**
*It’s no different to any other diagnostic test that we’d do really. We’re all fairly used to going through the process of consenting and counselling patients for diagnostic tests in a whole range of settings so it’s, I mean obviously clinical genetics is perhaps a bit more complex than some of the other tests, but by no means the most complex testing that we do. O5*
*I mean, you know, they’re seeing an oncologist to have oncology treatment, if we think it’s relevant to have BRCA testing, yes there is the issue of the implications for the rest of the family, but there’s direct implication for that patient’s management and we direct all the other things we need to know about that patient in order to direct their management so it seems odd to me for this one particular thing we have to refer them on to somewhere else and wait for them to be seen and all the rest of it. So it seems to me simpler just to do it in the context of the bit of the service or the bit of the pathway where the patient is being seen and where it’s directly relevant. O4*
**Redesigning the care pathway: Mainstreaming TFGT will streamline care**
*I think it will just speed up the testing process a bit because there’ll not be a, you know, there was a short delay while we had to wait for the clinical geneticist to see the patient, whereas now because they’re going straight to the test in our clinic that’ll just cut out that short delay. O5*
*The advantages of mainstreaming in oncology? I guess particularly just having control of that pathway and the results coming to me, I think it would just save time. O6*
**Relevance for practice: TFGT informs oncology practice**
*When we find a BRCA-positive patient it makes a significant difference to what we do, adding platinum, post-treatment post-surgical trial options at the moment. These come and go of course, and then obviously massive difference to the surgery. O3*
*So I will be seeking BRCA status in somebody who has got a breast cancer. Very different from somebody who’s at risk, primarily it’s because it will change their treatment, basically women with a BRCA 1, BRCA 2 germline mutation who have a breast cancer, their response rate to carboplatin is quite high, it’s higher than many other drugs. O4*

This group rarely pointed to their workload as a barrier to taking on the responsibility for offering and consenting patients for TFGT, indeed, like the clinical genetics team, they regarded mainstreaming in oncology as enabling them to offer breast cancer patients streamlined or expedited care. Finally, the oncologists regarded TFGT as having a great deal of relevance for their practice. They described genetic testing as allowing them to stratify their patients to ensure that individuals receive the most effective treatment. Thus, in contrast to the surgical team, this group saw TFGT as potentially facilitating their practice not constraining it.

In summary, the data suggest that different groups of healthcare providers perceive TFGT differently and suggest there are a number of barriers to the implementation of mainstreamed genetic services in some specialities. The following section outlines a number of ways in which these barriers might be overcome  (see Table 4 below).

**Table 4:** Moving genetic testing into the mainstream

**Maintain communication**
*I think the genetics services and the cancer services, I think we need a more cohesive approach, we need a better understanding of what each of us is thinking. CG3*
*We have quite a lot of informal, I mean mostly with name in clinical genetics team quite a lot of informal positive contact. But no, we live in slightly different worlds. Which is a shame O4*
*[I would like to] have an opportunity to sit down with the people who do that discussion now and make sure that my amateur version of it is covering the same ground…. I could do with like a little chat from the genetics service folk and maybe refreshers or an ongoing conversation from time to time to make sure we don’t diverge our approach as the service develops’. O3*
*I think mainstreaming could happen very quickly, it just requires us as a group of oncologists to sit down and have that other conversation. We don’t meet all of us terribly often because half of them do clinics outside so there aren’t many days a week when all of us, are actually here.…We have meetings about every three months and there always seem to be more pressing, urgent issues to resolve. I guess we just need to put it on the agenda for the next one. It’s just, it’s getting everybody in the room and agreeing, and having the conversation and people being comfortable. O4*
**Identify speciality champions**
*If someone is in a department, you know, in renal or something and they’ve got a particular interest in genetics then it makes a massive difference CG5*
*You need champions. I think all mainstreaming you need a champion in the mainstream specialty that wants to work with you, because only they really know how it fits with their way of working, their colleagues. We don’t know, we think we know what they want, and we have asked them, we keep asking them, we haven’t just invented it in a vacuum. I think you need to be in the specialty to understand the psychology of the specialty, really. And that’s strange. You know, our best, our best links with other specialties are when we’ve got a champion. CG4*
**Develop clear guidance**
*Work on any pathway, particularly something that’s probably got variable input is quite good to try and set it out clearly, particularly from the patient’s point of view, the patient expectation, so if you set out a clear pathway the patient then trundles along quite gently and quietly and knows what’s going on and you get less anxiety and less uncertainty and therefore less questioning and therefore less time taken up by the professional. If the pathway is well set out and well organized, I think generally you get less fall out from it which you as the consultant have to pick up. And I think from our point of view it’s better if there is a well set up pathway because then all the right people go into that pathway. So the more you know about, as a professional about the pathway and how it’s set out the better you use it basically. And if it’s a well thought out, well set up pathway then the patients will get the best out of it without causing more work. O1*
*I know they produce guidelines of what people we’ll refer,.. but I’ve been onto them clinical genetics team about this a number of times, that we lack very good genetic tools. There are some online assessment tools. But sometimes we could do, you know, there are apps for everything. We need better apps for genetic testing. You know, identifying which patients should be genetically tested. S2*

### Moving genetic testing into the mainstream

First, successful mainstreaming of genetics/genomics may depend upon the degree of inter-professional communication that exists at individual sites. Many of our interviewees commented that the layout of their hospital and the makeup of the MDMs at the study site, which the genetics team did not attend, meant that there was little contact between the different teams and this was perceived as impeding communication about mainstreaming. Members of the surgical and clinical genetics teams said they rarely met and did not know each other well, if at all, which may explain why members of the surgical team appeared to have unrealistic expectations of what they would be asked to do in a mainstreamed service, with a number suggesting they would be required to provide genetic counselling (e.g. S6, Table 2). Others suggested that communication about mainstreaming should be on-going between and within teams.

Beyond establishing effective communication about mainstreaming, it was observed that handing over TFGT to non-genetics specialists could be facilitated by the recruitment of mainstreaming champions, individuals from the target specialty who advocate for the implementation of genetic testing. The genetics team observed that the ovarian pathway at the study site had been mainstreamed a few years previously primarily because the gynaecological oncologists had lobbied for its implementation. They observed that a couple of the oncologists were promoting TFGT in the oncology clinic and thus, champions were emerging in the breast cancer pathway. Finally, a number of interviewees commented that effective implementation of mainstreaming could be facilitated by comprehensive guidelines detailing how patients should be managed. These would not only define the patient pathway, but also could be used to justify or reinforce particular patient management decisions.

## Discussion

This study suggests that non-genetics specialists involved in the provision of TFGT to newly diagnosed breast cancer patients have contrasting views about the mainstreaming of this service. These relate specifically to their perceptions of the roles and responsibilities related to their speciality—what a surgeon/oncologist does or should be normally expected to do—whether offering genetic testing would negatively or positively impact their workload and lastly, the perceived relevance of genetic information for patient care.

In contrast to earlier studies that suggest breast surgeons would be best placed to offer TFGT to newly diagnosed patients [20, 21] the breast surgeons we interviewed summarily dismissed this suggestion, citing a lack of expertise in providing genetic counselling and support for patients making this decision. The discrepancy between these findings may arise from the fact that, with the exception of one study [21] that involved non-genetics professionals who had previously offered TFGT during an RCT, this earlier research has involved healthcare professionals who were considering a hypothetical service. Arguably, our observations are better supported by a recent US study [22] of breast surgeons potentially responsible for offering TFGT and consenting patients, which found that that over two-thirds regularly refer patients on for genetic counselling and testing, citing a lack of confidence about discussing the individual and familial implications of genetic testing with patients.

The implementation of genetic/genomic testing in mainstream specialities has implications for workload management, and all three groups in this study discussed the impact of (potentially) offering TFGT on their workload [13, 14]. The surgical team suggested that they do not have the capacity to discuss TFGT with newly diagnosed patients, reinforcing earlier research, which found that over 40% of non-genetics professionals said that providing TFGT took more time and increased their workload [21] and reflects the findings of a recent systematic review, which suggests lack of consultation time is seen as a major barrier to incorporating genetics into primary care [25]. In contrast, the clinical genetics team and oncologists we interviewed supported mainstreaming commenting that this would simplify the patient pathway and expedite treatment decisions, similar views were expressed by non genetics professionals in Douma et al.’s study [21], with 90% perceiving the rapid turnaround time for test results as a major advantage of TFGT.

Finally, the perceived relevance of genetics/genomics for practice was an important influence on interviewees’ responses. Oncologists emphasised the utility of establishing patients’ *BRCA* status for treatment, providing further confirmation that mainstreaming is widely accepted in oncology [6, 15–17]. In contrast, the surgical team regarded TFGT as having little relevance for patient care in the short-term, constructing *BRCA* testing as primarily important for secondary prevention in the medium term [19]. The surgical team’s prioritisation of treatment rather than prevention may be influenced by the 16 week treatment targets issued by the Department of Health in the UK, which may have the effect of focussing this group on short-term goals. Similar observations were recorded in an evaluation of genetics pilots in the UK [26], which found that primary care practitioners were more concerned about meeting pre-existing governmental targets than introducing genetic services. The idea that non-genetics healthcare professionals may struggle to see the relevance of genetics for their practice has been observed in earlier studies, which suggested that General Practitioners’ ambivalence about the integration of genetics in primary care was linked to their lack of knowledge about genetics and their uncertainty about the relevance of genetic testing for patient management in primary care [25–27].

Finally, these interviewees pointed to a number of issues that may facilitate the implementation of genetic testing in the mainstream: more effective communication, particularly across specialities, identification of mainstreaming champions and more comprehensive guidelines/educational support for those in mainstream specialities [28, 29]. These suggestions confirm earlier observations made by those involved in piloting mainstreamed services in the UK [14] and Australia [21], and reflect earlier experiences at the study site.

### Limitations

This research has a number of limitations. First, data collection was limited to one site, thus restricting the study’s generalizability. Despite this, the findings confirm those generated in consultations [13] and questionnaire studies [20], which have ascertained hypothetical views about TFGT and mainstreaming. Second the data captured staff views about mainstreaming prior to the implementation of the service only; arguably a longitudinal evaluation of the implementation of mainstreamed services would have enabled us not only to identify potential barriers, but also to determine how these were overcome. We suggest that such an evaluation should be the focus of future research.

## Conclusions

If genetic/genomic testing is to be implemented in mainstream specialties, then we need to think strategically about where, and how to introduce this service and, more importantly, who will introduce it? With regard to the latter question, different specialists may have clear ideas about whether they have the expertise or capacity to provide this service and its relevance for their practice and these may thwart attempts at implementation. Mainstreaming may have the potential to streamline cancer care, but it can only do so if non-geneticists who work in the mainstream can see its potential, and this may be a challenge without further education of the healthcare workforce.
